# The influence of spontaneous activity on stimulus processing in primary visual cortex

**DOI:** 10.1016/j.neuroimage.2011.10.066

**Published:** 2012-02-01

**Authors:** M.L. Schölvinck, K.J. Friston, G. Rees

**Affiliations:** a Institute of Cognitive Neuroscience, University College London, 17 Queen Square, London WC1N 3AR, UK; b Wellcome Trust Centre for Neuroimaging, University College London, 12 Queen Square, London WC1N 3BG, UK

**Keywords:** Spontaneous activity, Resting-state functional connectivity, Primary visual cortex, Psycho-physiological interaction, fMRI

## Abstract

Spontaneous activity in the resting human brain has been studied extensively; however, how such activity affects the local processing of a sensory stimulus is relatively unknown. Here, we examined the impact of spontaneous activity in primary visual cortex on neuronal and behavioural responses to a simple visual stimulus, using functional MRI. Stimulus-evoked responses remained essentially unchanged by spontaneous fluctuations, combining with them in a largely linear fashion (i.e., with little evidence for an interaction). However, interactions between spontaneous fluctuations and stimulus-evoked responses were evident behaviourally; high levels of spontaneous activity tended to be associated with increased stimulus detection at perceptual threshold. Our results extend those found in studies of spontaneous fluctuations in motor cortex and higher order visual areas, and suggest a fundamental role for spontaneous activity in stimulus processing.

## Introduction

Traditionally, functional neuroimaging (fMRI) studies have focused on the brain's response to an external stimulus or task. Yet even at rest, fMRI signals display spontaneous fluctuations in activity. Many studies have examined the spatiotemporal structure of this so-called resting-state activity, identifying networks of functionally related areas that exhibit correlated activity (Fox and Raichle, 2007; Raichle, 2010). In contrast to the large body of research on resting-state functional networks, the properties of spontaneous fMRI activity under stimulus or task conditions have not been investigated extensively. In particular, the effect of spontaneous activity on stimulus processing remains unclear.

During stimulus processing, incoming stimuli evoke activity in sensory brain regions that are also subject to spontaneous fluctuations in the inputs from other brain regions. We will refer to these as spontaneous inputs and regard the resulting response of a stimulus-processing region as spontaneous fluctuations around an evoked response. These fluctuations reflect both the spontaneous input itself and its interaction with the evoked response. The nature of the interaction between spontaneous and stimulus-evoked input remains unclear. Although a couple of studies have shown that spontaneous and stimulus-evoked input are linearly additive in motor cortex (Fox et al., 2006) and occipital cortex (Arieli et al., 1996; Bianciardi et al., 2009), other studies in extrastriate visual cortex (Hesselmann et al., 2008a,b) have argued that they interact in a nonlinear way. Furthermore, the quantitative effect of spontaneous fluctuations on the variability in observed stimulus-induced responses has not been addressed in great detail. It is well-known that repeated presentation of identical stimuli leads to variable BOLD responses (Aguirre et al., 1998), and identical sensory input can give rise to qualitatively different percepts (Cusack, 2005; Schölvinck and Rees, 2010). Several lines of evidence suggest that spontaneous fluctuations play a role in this perceptual variability; specifically, activity in several brain areas just prior to a stimulus influences perception of bistable illusions (Hesselmann et al., 2008a), coherent motion (Hesselmann et al., 2008b), and faint somatosensory (Boly et al., 2007) and auditory (Sadaghiani et al., 2009) stimuli. However, despite the importance of spontaneous fluctuations for the sensitivity of fMRI to detect evoked responses, the effect of spontaneous input on the variability in the evoked BOLD response itself has only been investigated in motor cortex (Fox et al., 2006, 2007) and is unknown for other brain areas.

When a stimulus evokes activity in a particular brain region, spontaneous activity in this region cannot be measured directly (because activity ceases to be spontaneous). However, this region still receives inputs from other regions showing spontaneous fluctuations. A common way to estimate spontaneous inputs to a particular brain region, therefore, is to use the activity in such functionally connected regions (that do not show evoked responses) as a proxy for spontaneous inputs that would have been observed in the absence of evoked responses. For example, in motor cortex spontaneous activity in right motor cortex has been used as a proxy for spontaneous inputs to the left motor cortex (Fox et al., 2006, 2007). A central issue in the use of such proxies is the assumption that spontaneous input remains the same under rest and activation. This is because the proxy region is chosen based on its correlation with the stimulus region *at rest*, after which its activity is used as a proxy for spontaneous input to the stimulus-responsive region *during the task*. Here, we attempted to resolve this issue by treating the proxy and stimulus as separable causes of the activity in a stimulus-sensitive region. If stimulus processing changes the region's sensitivity to spontaneous inputs, then we expect to see an interaction between the proxy and the stimulus when explaining observed fluctuations about evoked responses in the stimulus region.

We therefore investigated the influence of spontaneous activity on stimulus processing in human primary visual cortex (V1). Several lines of evidence suggest that activity in V1 does not merely reflect sensory input per se, but interacts with endogenous factors including attention (Kastner et al., 1999) and perceptual decisions (Ress et al., 2000; Ress and Heeger, 2003). The combination of clear activity modulation by stimuli as well as by endogenous factors, make V1 an ideal candidate for studying how internally generated (spontaneous) input interacts with externally evoked (stimulus-induced) activity.

Using a novel method for choosing a proxy of spontaneous activity, we evaluated the influence of spontaneous activity on stimulus processing in visual cortex and compared this to earlier findings in motor cortex (Fox et al., 2006, 2007). We focused on two key issues; the interaction between spontaneous and stimulus-evoked inputs during neural processing of stimuli, and the influence of spontaneous fluctuations on behaviour. Our results support previous findings in motor cortex; namely, a simple linear summation of spontaneous and stimulus-evoked inputs predominated over their interaction. Crucially, however, the spontaneous fluctuations were associated with differences in task performance. We discuss the implications of this for understanding the role of spontaneous activity more generally in the human brain.

## Methods

### Observers and stimuli

**Fig. 1:**
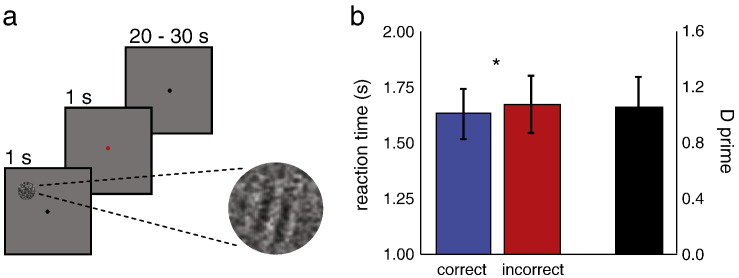
Task and behavioural data. (a) Participants were shown a small circle in their left upper visual field, which consisted of noise or noise plus an embedded low contrast grating (shown here; the grating has higher contrast than in the actual experiment for illustration purposes). The stimulus was shown for 1 s, after which participants had 1 s to respond (indicated by the fixation dot turning red). Inter-stimulus intervals were 20–30 s. (b) Average reaction times (left) and D-primes (right) for all participants.

Six healthy volunteers with normal vision (22–35 years old; 3 females) gave written informed consent to participate in the experiment, which was approved by the local ethics committee. They viewed a low-contrast (luminance ~ 25.5 cd/m^2^) stimulus on a uniform grey background (luminance ~ 28.4 cd/m^2^) (Fig. 1a). The stimulus was a circle composed of dark and light grey random (spatiotemporally white) noise, on which a low-contrast Gabor-grating (frequency: 0.93 cycle/degree) was superimposed in half the trials. There was no detectable difference in luminance between the random noise alone and the noise plus Gabor-grating, as measured with a photometer. The stimulus was displayed in the upper left visual field at 7.5° eccentricity (4.0° up, 6.4° across) and was 4.0° in diameter. Observers were instructed to attend to a central fixation dot while the stimuli were presented briefly (for 1 s), after which the fixation dot turned red to indicate that a response was required. Participants responded with their right hand using a keypad; they were required to press either the right button (grating present) or the left button (grating absent) within a one second response window. The inter-stimulus interval (ISI) was randomised to minimise anticipation; the next stimulus followed after 20–30 s.

### FMRI data acquisition

A 3T Allegra MRI scanner (Siemens Medical Systems, Erlangen, Germany) with a standard transmit–receive head coil was used to acquire functional data with a single-shot gradient echo isotropic echo planar imaging (EPI) sequence (matrix size 64 × 72; field of view 192 × 216 mm; in-plane resolution 3 × 3 mm; 32 slices in descending acquisition order; slice thickness 2 mm with a 1 mm gap; echo time 30 ms; acquisition time per slice 60 ms; TR 1920 ms). Scanning parameters were similar for functional and retinotopy runs (see below). In a separate session, an anatomical T1 weighted (Mdeft) image was acquired (matrix size 256 × 240; field of view 256 × 240 mm; in-plane resolution 1 × 1 mm; 176 sagittal slices of 1 mm thickness, no gap; echo time 2.4 ms; acquisition time per slice 7.92 ms). Each functional run of the main experiment comprised 180 volumes. Each subject completed 9–12 functional runs, acquired in two separate sessions. In the middle of each session, double-echo FLASH images with TE1 10 ms, TE2 12.46 ms, 3 × 3 × 2 mm resolution, and 1 mm gap were acquired. These fieldmaps were used to correct geometric distortions in the EPI images due to field inhomogeneities. During scanning, respiration volume and cardiac pulse were measured using a breathing belt placed round the participant's waist and an MRI compatible pulse oximeter (Model 8600 F0, Nonin Medical, Inc. Plymouth, MN) attached to the participant's finger. These data, together with scanner slice synchronisation pulses, were sampled using the Spike2 data acquisition system (Cambridge Electronic Design Limited, Cambridge, UK) and used for physiological noise correction (see below). Eye position was continually sampled at 60 Hz using an ASL 504 LRO infrared video-based MRI compatible eye tracker (Applied Science Laboratory, Bedford, MA). For four participants, half of the eye data were not recorded due to technical difficulties. Eye movements were defined as variance round the mean (x,y) position of the eye. Statistical analysis was performed to confirm stable fixation throughout the experiment.

### Procedure

**Fig. 2:**
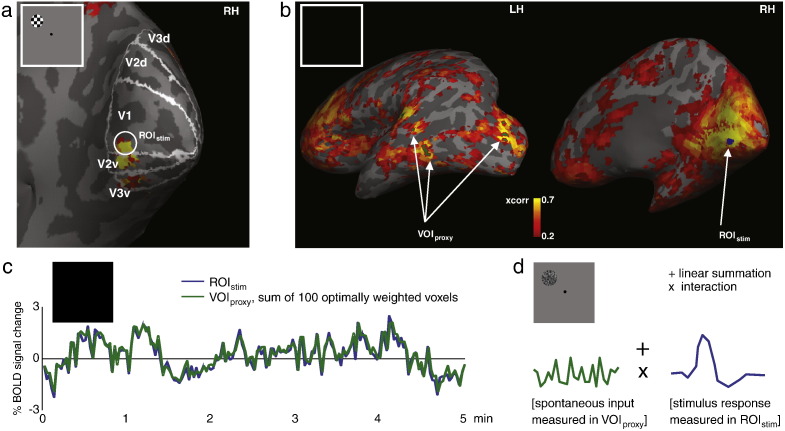
Overview of analysis. (a) Combining activity evoked by a stimulus localiser (inset) with the borders of the visual areas revealed by retinotopic mapping defined retinotopic regions in V1, V2, and V3 representing the spatial location of the stimulus. The stimulus location in V1 (encircled) was chosen as ROI_stim_. RH, right hemisphere. (b) The time course of ROI_stim_ (blue) during the rest run (inset) was correlated with all other voxels in the brain, and the 100 most correlated voxels (after excluding the voxels significantly activated by the stimulus) were collectively termed the VOI_proxy_, shown here in green. LH, left hemisphere. (c) The time courses of the 100 voxels in VOI_proxy_ (green) were independently weighted and then summed to give the best possible estimate of the time course of ROI_stim_ (blue) during the rest run (inset). On average, the activity in VOI_proxy_ explained 60.6% of the variance in ROI_stim_; please note, however, that the close correspondence between the time courses of VOI_proxy_ and ROI_stim_ is shown here purely to illustrate the fMRI analysis, and does not influence the main analysis in any way, since the weights of the VOI_proxy_ voxels were applied to independent data. (d) The VOI_proxy_ was then used during the task runs (inset) to determine the effect of spontaneous inputs measured in VOI_proxy_(left) on stimulus-evoked BOLD responses measured in ROI_stim_ (right), distinguishing between either simple linear summation (+) or a more complex interaction (x).

Participants were tested before scanning to ensure they could assign consistent responses to the different stimuli (noise and noise + grating). Their performance was held constant at 75% correct (D-prime ~ 1) by means of a staircase procedure; the range of contrasts used for the experiment was 3.5–6%. They performed a further staircase block in the scanner before fMRI data acquisition. The contrast of the grating was individually set to the level determined by the staircase procedure, and kept constant at this level throughout the entire fMRI experiment. Subsequently, they completed between 9 and 12 (six minute) runs of scanning, acquired in two separate sessions. A run started with instructions for the button presses on the screen, then a 10 second blank, followed by a stimulus every 20–30 s. In the middle of the first session, a rest run was acquired during which the participants were instructed to close their eyes and relax for 6 min. This run was used to estimate resting-state fluctuations (see below). In between the functional runs, participants were given two runs of a localiser stimulus, comprising a black-and-white checkerboard stimulus (flickering at 10 Hz on a grey background) of identical size and location as the stimulus in the task. These runs were used to localise stimulus-sensitive regions in V1, V2, and V3 (Fig. 2a; Supplementary materials). Standard retinotopic (meridian) maps and T1-weighted structural scans were acquired in a third session.

### Data analysis

Functional data were analysed using SPM5 (www.fil.ion.ucl.ac.uk/spm/software/spm5/). The first 5 images of each run were discarded to allow for T1 equilibration. Preprocessing of the data involved realignment of each scan to the first scan of the first experimental run, correction of slice time acquisition differences, coregistration of the functional data to the structural scan, normalisation to the MNI template brain, and smoothing by a 6 mm Gaussian kernel. The data were filtered with a standard 128-s cut-off, high-pass filter to remove low-frequency noise including differences between runs, while at the same time preserving as many of the spontaneous fMRI fluctuations as possible (Cordes et al., 2001). Physiological data (respiration and heart beat) were modelled using an in-house developed Matlab toolbox (Hutton et al., 2011) based on RETROICOR (Glover et al., 2000). A time series representing the change in respiration was calculated from the mean respiratory waveform by taking the standard deviation at each time point over a 6-second sliding window, and then convolving with the so-called respiratory response function (Birn et al., 2008). The resulting respiration volume per unit time (RVT), and basis sets of sine and cosine Fourier series components extending to the 5th harmonic (i.e. 10 regressors) for the cardiac phase and 3rd harmonic (i.e. 6 regressors) for the respiratory phase were used to model the physiologic fluctuations. This resulted in a total of 17 regressors, which were sampled at the slice in each image volume that covered most of the stimulus region in V1 (typically slice 23 out of 32) to give a set of values for each TR. The resulting regressors were included as confounds in the first level analysis for each participant. Movement parameters in the three directions of motion and three degrees of rotation were also included as confounds. Regressors modelling the stimulus were formed by convolving a stick function with a canonical hemodynamic response function. Regressors modelling spontaneous effects and the interaction with the stimulus were constructed using custom code, written in MATLAB (http://www.mathworks.com) (see below). The regressors modelling the stimulus, the proxy for spontaneous inputs, their interaction, and the nuisance regressors were used to perform a random effects analysis over participants in the usual way. This entailed estimating (contrasts of) parameters encoding the effects of interest using a standard linear convolution model at the first, within-subject, level (over all runs) and then passing the resulting contrast images to one sample *t*-tests at the second, between-subject, level. The resulting statistical parametric maps (SPMs) were then used to test for the main effects of stimulus, spontaneous input and interaction. The statistical significance of these effects was assessed within a sphere of 8 mm radius centred on the voxel in V1 showing the highest activation to the localiser stimulus.

### Optimising the proxy of spontaneous activity

**Supplementary Fig. 1:**
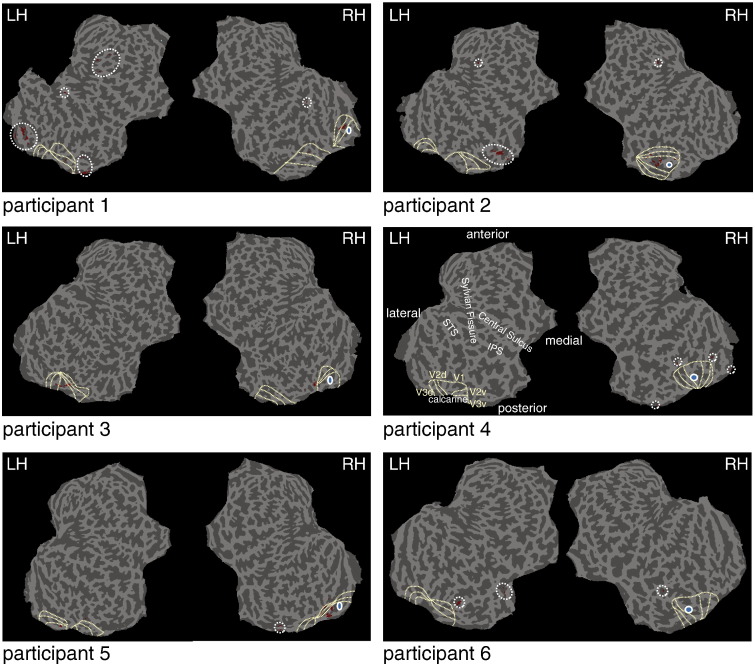
Location of VOI_proxy_. The location of the 100 voxels best correlated to the stimulus region during rest is shown for all participants. VOI_proxy_ voxels are in red and encircled with white broken lines, the location of ROI_stim_ is in blue and encircled with a solid line. Outlines of visual areas V1, V2, and V3 are shown on each hemisphere with light yellow broken lines. Labels belonging to these visual areas are shown on the left hemisphere of one participant showing no VOI_proxy_ voxels; these labels hold for all (left) hemispheres shown and are mirrored in the opposite (right) hemispheres. In some participants, visual areas are split up by the way the cortex is cut and flattened. The one hemisphere showing no VOI_proxy_ voxels also shows several anatomical landmarks. STS, superior temporal sulcus. IPS, inferior parietal sulcus. LH, left hemisphere. RH, right hemisphere.

**Supplementary Fig. 2:**
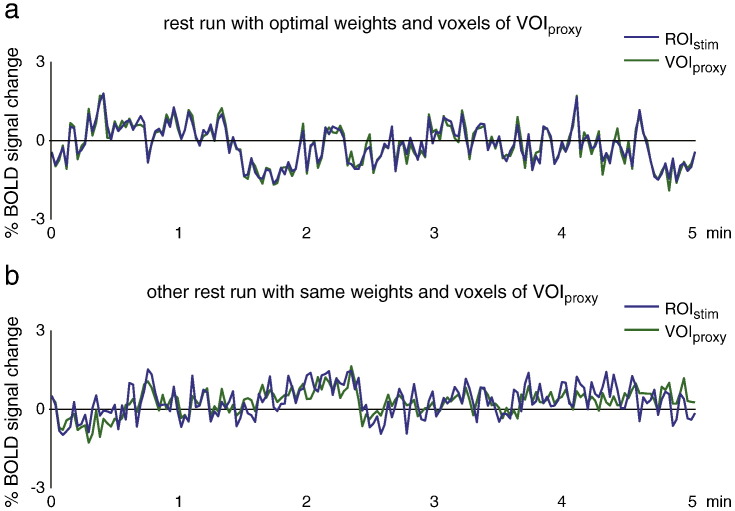
Stability of coupling between VOI_proxy_ and ROI_stim_. (a) The time course of ROI_stim_ during the rest run for one participant is shown together with the average time course of VOI_proxy_. The voxels of this VOI_proxy_ and their weights were chosen such as to maximise the correspondence with the time course of ROI_stim_, as explained in the main text. (b) The time course of ROI_stim_ during another rest run in the same participant, collected on a different day. The same voxels and weights were used to construct the average time course of VOI_proxy_, and it is evident that there is still a close correspondence between the two time courses, confirming the stability of the coupling between ROI_stim_ and VOI_proxy_ over time. In (a), the activity in VOI_proxy_ explained 93.7% of the variance in ROI_stim_, in (b) this was 46.4%.

The proxy for spontaneous inputs to the V1 stimulus region was acquired from the rest run data. After pre-processing these data, each voxel time course was converted into units of percent change by subtracting, and then dividing by the mean of its time course. Data were high-pass filtered at 0.005 Hz (Cordes et al., 2001) and any activity correlated with motion, cardiac and respiratory cycle (see above), and the average time course across all voxels (the ‘global signal’) were removed by linear regression. This suppressed artifactual and global (regional nonspecific) correlations in the data and ensured that the proxy was largely neural in origin. Note that the effect of this on correlations among global modes of activity (Murphy et al., 2009) is not an issue in the present study. To estimate the spontaneous inputs to V1, we used the spontaneous activity in remote regions showing coherent fluctuations with the V1 stimulus region. The average time course of the stimulus region in V1 (ROI_stim_) during the rest run was used as a seed region to compute the correlation with the time courses of all other voxels in the brain (Biswal et al., 1995; Fox et al., 2006) (Fig. 2b). The resulting correlation map was masked with an SPM of stimulus-evoked responses (p < 0.05, FWE corrected) to exclude any voxels that were activated by the stimulus. Of those voxels that remained, the time courses of the 100 voxels with the highest correlation were used to find the optimum weights β that furnished a least squares estimate of the activity in the V1 seed region (Fig. 2c):

(1) $$F=\beta_{1}f_{1}+\beta_{2}f_{2}+\ldots +\beta_{100}f_{100}+\varepsilon$$

where *F* is the (mean-corrected and adjusted) time course of the stimulus region (ROI_stim_), *f*_*1*_*…f*_*100*_ are the time courses of the correlated voxels, *β*_*1*_*…β*_*100*_ are their weights and *ε* is the error. The 100 correlated voxels together were termed VOI_proxy_ (since these are distributed and non-contiguous voxels we chose to call them a Volume Of Interest, as opposed to a Region Of Interest), and their weighted average time course served as our proxy for spontaneous inputs to V1. Note that these weights were used only to construct the proxy and are not an estimate of the 100 voxels' contribution to V1 activity during stimulus processing. For all participants, most of the 100 voxels were located in bilateral occipital cortex (see Supplementary Fig. 1 and Supplementary Table 1 for the actual location of the 100 voxels for each participant). Applying the same weights to the same VOI_proxy_ voxels in two rest runs collected on separate days in one participant confirmed that the coupling of VOI_proxy_ to ROI_stim_ was relatively stable over time (Supplementary Fig. 2).

### Testing for an interaction between spontaneous and stimulus inputs

To investigate a possible interaction between spontaneous and stimulus inputs (Fig. 2d), we modelled the data using a standard linear convolution model in which both the stimulus and the spontaneous inputs were used as explanatory variables:

(2) $$Y=\beta_{1}X+\beta_{2}E+\beta_{3}S+\beta_{4}\left(E*S\right)+\varepsilon$$

The first term *X* is a partition of the design matrix containing a constant (and the other confounds). The second and third terms model hemodynamic activity evoked by the stimulus (*E*) and spontaneous inputs (*S*). The latter corresponds to the activity in VOI_proxy_ ; the activity during the task in the VOI_proxy_ voxels which were selected based on their functional connection with ROI_stim_ at rest (note that there is no need to convolve the spontaneous input with a hemodynamic response function because it is already a hemodynamic measure). The fourth term models the interaction between evoked and spontaneous inputs. This analysis is called a psychophysiological interaction (PPI) analysis and is similar to that employed by Bianciardi et al., 2009. Tests for the main effects of spontaneous and evoked influences and their interaction were based on the appropriate contrast images from a first (within-subjects) level analysis. These contrast images were analysed using one sample *t*-tests at the second (between-subjects) level to produce random effects SPMs of the two main effects and their interaction. Statistical significance was assessed using random field theory corrected *p*-values, within an 8-mm sphere centred on the voxel in V1 showing the highest localiser activation. Effect size of the interaction was assessed by computing posterior probability maps at the second (between-subjects) level, to show with 95% probability all voxels whose activation, due to the interaction effect, exceeded several (increasing) activation thresholds.

In a second PPI analysis, we modelled spontaneous inputs together with task-related activity (task vs rest) by including both task and rest runs in the design. The interaction term in this PPI reveals regions whose functional connectivity to the stimulus region changes depending on the task, and therefore assesses the constancy of the coupling between VOI_proxy_ and ROI_stim_ under stimulus and rest conditions. This PPI was analysed in a similar way as the main PPI analysis.

### Quantifying the variance explained by spontaneous activity

Following the main PPI analysis, we proceeded to quantify the variance explained by spontaneous input by computing the reduction in BOLD fluctuations (‘noise’) about the average stimulus-evoked response (‘signal’) and the resulting change in signal-to-noise ratio (SNR). The individual BOLD response time courses to the stimulus, as well as the average BOLD response, were plotted for ROI_stim_, and the signal power, noise power, and SNR calculated. Signal power was computed as the mean squared deviation of the average BOLD response from baseline; noise power was computed as the mean squared deviation of the residual (individual BOLD response−average BOLD response). For each participant individually, the weighted average time course from VOI_proxy_ was subtracted from the ROI_stim_ time course. The resulting ROI_stim_ time course was called the ‘corrected’ ROI_stim_ time course. The individual and average BOLD responses to the stimulus were plotted from the corrected ROI_stim_ time course and again the signal power, noise power, and SNR were calculated. These values were compared to the original values (before subtracting the VOI_proxy_ time course). This procedure is similar to that employed by Fox et al., 2006.

Statistical comparisons of the measures of noise power, signal power, and SNR were done at two different levels. The first level was a within subject comparison in which the noise power was computed for each individual trial. This allowed us to determine whether correcting for spontaneous activity led to a significant reduction in noise or a significant improvement in SNR for an individual participant. The second statistical analysis was at the group level. Here the noise power was averaged across trials for each individual, and it was determined whether there was a significant effect of correction for spontaneous activity on signal power, noise power, or SNR at the group level. For both within subject and group level analyses, significance was assessed using a Wilcoxon paired nonparametric test. Similar analyses were performed for ROI_stim_ in extrastriate areas V2 and V3, using separately computed VOI_proxy_'s.

### Analysis of the effect of spontaneous activity on behaviour

Finally, we examined the relationship between spontaneous fluctuations and the behavioural decision that the participant made based on the stimulus. We divided the trials into those which had elicited a correct or incorrect response to the presence of the grating. We then examined the mean BOLD response on correct and incorrect trials and tested for a significant difference in observed responses. Given that spontaneous fluctuations were dominated by spontaneous inputs (relative to any interaction; see Results), we quantified the effect of spontaneous inputs on behaviour by dividing trials into two equally sized sets (per participant) with low and high average levels of spontaneous input as estimated by VOI_proxy_. We then computed the probability of a correct response for each of the two trial sets for each participant.

For details on the retinotopic mapping and ROI localiser runs and their data analysis, please see Supplementary materials.

## Results

Six healthy volunteers were asked to detect a low-contrast grating, present in half the trials, amidst a circular patch of random grey noise in their left upper visual field. Stimuli were presented briefly (1 s) followed by long inter-stimulus intervals (20–30 s). On average, participants completed 128 trials in 9–12 six-minute runs of the task. Participants' average D-prime was 1.06, which indicates that the grating was presented at perceptual threshold (Fig. 1b). The average reaction time to the stimulus was 1643 ms measured from stimulus onset; since participants were allowed to respond only after termination of the 1-second long stimulus, this reaction time was relatively long. Reaction times were slightly shorter for correct than for incorrect trials (Fig. 1b) (1619 ms and 1660 ms respectively, t(5) = 4.49, p = 0.007). For individual participant data, please see Supplementary Table 2.

### Spontaneous and stimulus-evoked influences summate linearly

**Supplementary Fig. 3:**
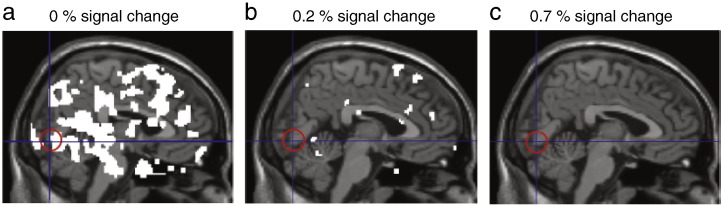
Interaction effect size. Posterior probability maps show all voxels whose activation by the interaction of spontaneous and stimulus-induced inputs exceeds, with 95% probability, a threshold of 0% BOLD signal change (a), 0.2% BOLD signal change (b), or 0.7% BOLD signal change (c). As can be readily observed, the interaction effect in the stimulus region in V1 (red circle), if any, is extremely small.

**Fig. 3:**
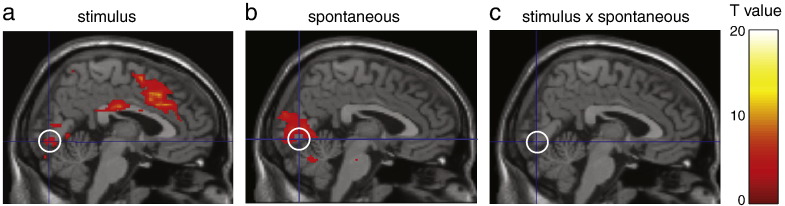
Linear superposition of spontaneous and evoked activity. (a) Brain responses explained by the stimulus. Please note the significant activation of the stimulus region in V1 (white circle), as well as motor areas related to the button presses in response to the stimulus. (b) Brain responses explained by the (proxy for) spontaneous input. As expected, the activity in VOI_proxy_ accounted for a significant fraction of the variance in visual cortex. (c) Brain responses explained by an interaction between stimulus-evoked and spontaneous activity. All SPMs are one-sample *t*-tests, thresholded at p < 0.001 (uncorrected), while statistics concerning the stimulus-sensitive region are reported at p < 0.05, FWE-corrected. Cross-hairs intersect at the location of ROI_stim_ in V1, MNI coordinates [2 − 78 − 8].

We first assessed the interaction between spontaneous activity and activity induced by stimuli during stimulus processing, using the activity in VOI_proxy_ as an estimate for the spontaneous fluctuations in ROI_stim_ (see Methods). To assess the relative influence of spontaneous and stimulus-evoked effects on the stimulus V1 region, we analysed its activity using a general linear model, in which both effects and their interaction were entered as explanatory variables (see Methods). This psychophysiological interaction (PPI) analysis assessed how well stimulus and spontaneous effects (and their interaction) could explain the observed BOLD responses. The main effect of stimuli revealed expected activations in visual and motor cortex, due to the visual stimulation and button presses in response to the stimuli (Fig. 3a and Table 1). Spontaneous activity in VOI_proxy_ accounted for a significant amount of variance in visual cortex, including the stimulus-sensitive region (Fig. 3b and Table 1). The relative contributions (explained variance) of stimulus and spontaneous effects in visual areas were 74.8% and 25.2%, respectively. Crucially, the PPI analysis showed that a linear mixture of spontaneous and stimulus-induced effects explained much more variance than any nonlinear interaction between the two; indeed, there was no discernable evidence for an interaction (Fig. 3c and Table 1), despite the fact our design was sufficiently empowered to show significant main effects. The lack of evidence for this interaction does not mean it is not there, simply that we failed to detect it with our present design. However, we showed that this interaction, if present, is quantitatively small in relation to the main effects of spontaneous and stimulus-induced effects by computing posterior probability maps showing the interaction effect size (Supplementary Fig. 3). We therefore conclude that spontaneous and stimulus-evoked input combined at least primarily additively to produce the observed BOLD responses, in any case in the current paradigm and brain area under study.

The absence of an interaction between spontaneous and stimulus-evoked input implies that the influence of spontaneous activity on the stimulus region remained largely unchanged under stimulus processing, and therefore that the coupling between the stimulus region and VOI_proxy_ is likely to be similar under stimulus and rest conditions. We confirmed this in a second PPI analysis, in which we included both task and rest runs and replaced the stimulus-effect variable with a task-effect variable (task vs. rest). The interaction term in this PPI analysis reveals regions whose coupling to the stimulus region changes depending on the presence of the task (relative to rest). We established that the influence of the spontaneous proxy on V1 did not change during task performance; i.e., the interaction between the psychological factor (task vs. rest) and the physiological factor (VOI_proxy_ activity) was not significant at p < 0.001 (uncorrected). This suggests that the 100 voxels provided a stable proxy for spontaneous inputs to the stimulus region in V1 during stimulus processing. To further confirm the similarity in coupling between VOI_proxy_ and the stimulus region under stimulus and rest conditions, we repeated the original analysis with a VOI_proxy_ computed from a rest run where participants were asked to fixate a fixation dot on a blank (grey) screen rather than lie in darkness with their eyes closed. This analysis showed very similar results to the results reported for the main PPI analysis.

**Supplementary Fig. 4:**
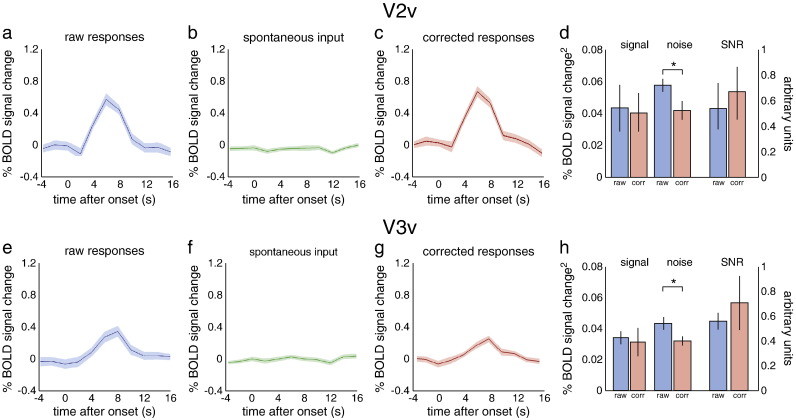
Effect of spontaneous activity on BOLD response variability in V2 and V3. (a,e) The raw BOLD response is plotted for all trials of the same representative participant as in Fig. 3. (b,f) The spontaneous input measured in separately computed VOI_proxy_'s for V2v and V3v. (c,g) Subtracting the spontaneous input reduced the variability in the evoked BOLD responses. (d,h) Signal power (left), noise power (middle), and SNR (right) in V2v and V3v for all participants before (‘raw’; in blue) and after (‘corr’; in red) subtracting the estimated spontaneous activity. The y-axis on the left in both graphs corresponds to the signal and noise power, the y-axis on the right to the SNR. Noise power was significantly reduced in both cases, yet SNR not significantly increased, after spontaneous activity subtraction. For details, see Fig. 3.

**Fig. 4:**
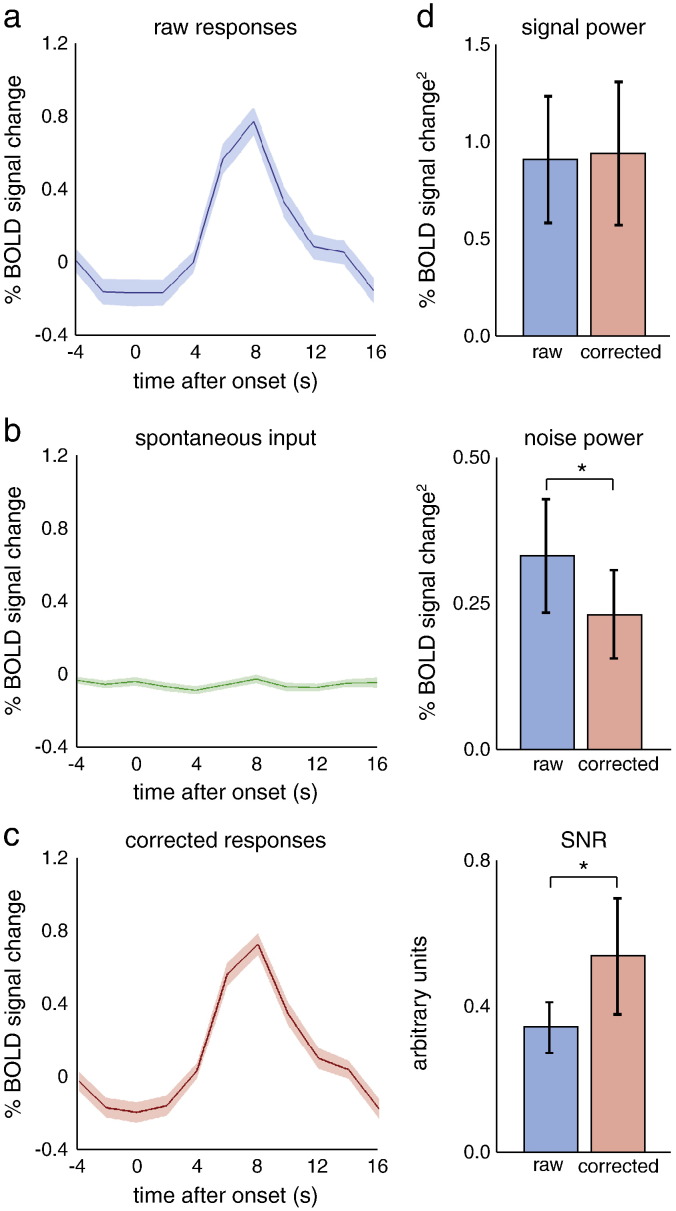
Effect of spontaneous activity on response variability in V1. (a) The raw BOLD response is plotted for all trials of a representative participant. Solid line shows the average; shaded area represents the standard error. (b) The spontaneous input into ROI_stim_ as estimated by VOI_proxy_. Solid line and shaded area, same as in a. (c) Subtracting this estimated spontaneous activity reduced the variability in the evoked BOLD responses. Solid line and shaded area, same as in a. (d) Signal power (top), noise power (middle), and SNR (bottom) for all participants before (left, in blue) and after (right, in red) subtracting the estimated spontaneous activity. Noise power was significantly reduced, and SNR significantly increased, after spontaneous activity subtraction.

To illustrate the quantitative effect of spontaneous inputs on BOLD responses in V1, we computed the mean and variance of the observed responses over trials (Fig. 4a). As established above, these BOLD responses can be explained largely as a linear mixture of stimulus-evoked activity and spontaneous input. Subtracting the (estimated) spontaneous input (Fig. 4b) from the responses in the stimulus region reduced the variability in the BOLD responses over trials substantially (Fig. 4c). This reduction corresponds to the variance explained by the (very significant) main effect of spontaneous activity reported above. For the representative participant shown in Fig. 4, the reduction in variability (attributable to spontaneous fluctuations or ‘noise’) was 25.4%. The average response (shown as a darker line in Figs. 4a–c) did not change when spontaneous fluctuations were subtracted, as demonstrated by a change in signal power of 0.0% for this participant. The resulting change in signal to noise ratio (SNR) was 34.0%. Similar reductions in variance and corresponding increases in SNR were obtained for all participants (Supplementary Table 3). Overall, response variance decreased by 25.5%, which implied a 32.2% increase in SNR (with an insignificant decrease in average response to the stimulus of 1.7%) (Fig. 4d). The BOLD responses in areas V2 and V3 exhibited a similar degree of variance reduction (although the corresponding SNR increase failed to reach significance) when adjusting for spontaneous effects (approximated by their respective, individually computed VOI_proxy_'s for V2 and V3), although the overall response was slightly lower than in the primary visual cortex (Supplementary Fig. 4).

### Spontaneous activity is related to stimulus perception

**Supplementary Fig. 5:**
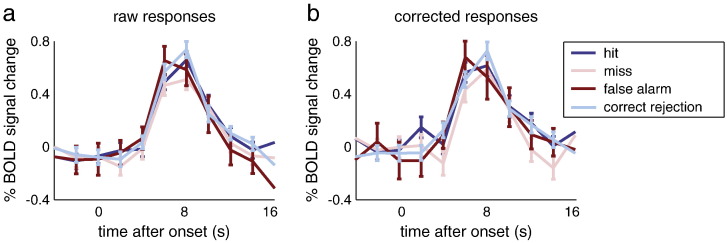
BOLD responses associated with four response categories. Dividing the trials into hits, misses, false alarms, and correct rejections did not reveal any differences in BOLD activity, either before (a) or after (b) subtracting spontaneous activity.

**Supplementary Fig. 6:**
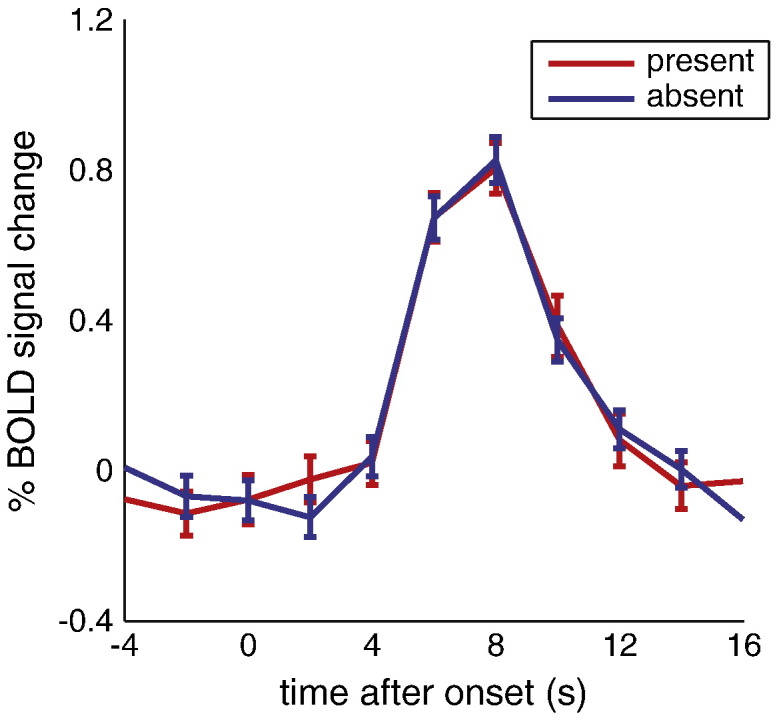
BOLD responses to the stimulus. BOLD responses were similar for those trials where the grating was present versus absent. Note that the circular patch of random grey noise was present in both cases, giving rise to the robust BOLD response even when the grating was absent.

**Fig. 5:**
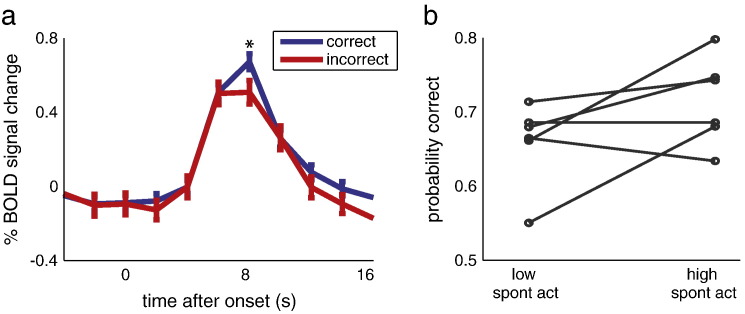
Effect of spontaneous activity on behaviour. (a) Dividing the trials into correct and incorrect trials revealed significantly higher peak activity for correct versus incorrect trials. (b) Dividing the trials into those with low versus high spontaneous input resulted in a higher probability correct for high spontaneous input trials, for almost all participants. Spont act, spontaneous activity.

To investigate the potential role of spontaneous fluctuations about the evoked response in V1 on stimulus perception, we classified the BOLD responses based on the participants' perceptual decisions on the stimuli (correct versus incorrect trials). This analysis revealed significantly higher activity for correct trials compared to incorrect trials at the peak of the BOLD response (Fig. 5a) (t(5) = 2.09, p = .033). Note that both correct and incorrect trials reflected a mixture of stimuli where the grating was present and absent (i.e. correct trials reflected hits and correct rejections, and incorrect trials reflected false alarms and misses), and this analysis thus may potentially conceal differences in BOLD responses under these four response categories (Ress and Heeger, 2003). While our study collected a large amount of data, due to the necessary spacing between trials (in order to accurately measure the entire BOLD response to the stimulus) we were not able to collect thousands of trials as was done in such earlier work (Ress and Heeger, 2003). Consequently, further subdividing the data into hits, misses, false alarms, and correct rejections did not yield significant differences in peak BOLD responses (Supplementary Fig. 5). However, the higher peak activity in correct compared to incorrect trials cannot have been due to the grating being present more often in the correct trials; in fact, the opposite was true (P(grating present | response correct) = 0.4237, while P(grating present | response incorrect) = 0.6944, t(5) = 3.15, p = .026). Furthermore, BOLD responses to the grating present versus absent, regardless of perceptual outcome, did not show any differences (Supplementary Fig. 6). Therefore, this difference in peak activity between correct and incorrect trials is not due to any differences in perceptual input, and can therefore be attributed to spontaneous fluctuations. Such spontaneous fluctuations might reflect many different processes, such as fluctuations in attention, arousal, hemodynamic changes of non-neuronal origin, and slow, spontaneous fluctuations in neural input. Given that BOLD response variability is partly due to spontaneous input (see above), one would expect to see differences in behaviour when comparing trials with high and low spontaneous input. In order to quantify the influence of spontaneous input on perceptual variability, we divided the trials into those where the average level of spontaneous input was low versus high (see Methods), and calculated the respective probability of a correct response. For most participants, the probability of being correct was higher when spontaneous activity was high (Fig. 5b), with an increase from about 65% correct to 72% correct (averaged over participants), although this effect was not significant. Collectively, these results suggest that spontaneous fluctuations partly account for differences in perception, implying that spontaneous input interacts with stimulus processing at the behavioural level; even if this interaction is small when measured in terms of hemodynamic responses.

## Discussion

The influence of spontaneous fluctuations in the fMRI BOLD signal on stimulus processing is an important but relatively unexplored area. Earlier work has shown that spontaneous fMRI activity can either add linearly to (Arieli et al., 1996; Fox et al., 2006) or interact with (Hesselmann et al., 2008a,b) activity evoked by stimuli, as well as influence perception (Boly et al., 2007; Sadaghiani et al., 2009). However, the specific effect of spontaneous activity on trial-to-trial variability in stimulus responses is largely unknown. Here, we investigated the impact of spontaneous activity on the processing of a simple visual stimulus in retinotopic primary visual cortex, using a new method for estimating the spontaneous influences on that region (a proxy comprising the weighted contribution from functionally connected but stimulus-insensitive voxels). We showed that spontaneous influences remained largely unchanged when a stimulus was shown compared to rest conditions, and did not change (interact with) stimulus-driven activity. Furthermore, spontaneous fluctuations about the (average) evoked response accounted for a large portion of both neuronal and behavioural variability in stimulus responses.

### Spontaneous activity under stimulus and rest conditions

A common assumption of studies that use a proxy region to estimate the spontaneous input to a stimulus region is that their influence remains the same under rest and task conditions. However, several studies show that the characteristics of spontaneous activity in visual cortex might change depending on behavioural state (Bianciardi et al., 2009; McAvoy et al., 2008; Nir et al., 2006; Yang et al., 2007). More specifically, the amplitude of spontaneous fluctuations is significantly reduced under eyes-open conditions compared to when the participant's eyes are closed (Bianciardi et al., 2009; McAvoy et al., 2008); an effect which has been attributed to visual imagery when the eyes are closed (Wang et al., 2008). Moreover, visual areas that are functionally unrelated exhibit a high degree of correlation during rest, but become decoupled during visual stimulation (Nir et al., 2006). These observations reflect the fact that functional connectivity subsumes correlations due to spontaneous fluctuations as well as stimulus-evoked activity. However, it could also reflect a true change in coupling (effective connectivity) that is induced by the task or stimulus. To disambiguate these explanations, we tested for the interaction between the two inputs into our stimulus region in V1: spontaneous fluctuations as measured by the proxy, and the presence of the stimulus.

Quantitatively speaking, spontaneous activity did not interact with the activity evoked by our stimulus; that is, spontaneous and stimulus-driven effects seemed to be linearly superimposed. FMRI studies of motor cortex (Fox et al., 2006) and visual cortex (Bianciardi et al., 2009) support these findings. However, based on quantitative differences in the BOLD responses associated with the two perceptual outcomes in Rubin's ambiguous face/vase illusion (Hesselmann et al., 2008a) or moving random dot stimuli at coherence threshold (Hesselmann et al., 2008b), other authors have argued that spontaneous activity in FFA and V5/MT interacts with neuronal processing evoked by the stimulus in a nonlinear way. At the behavioural level, we support these findings by showing that correct detection trials tended to be associated with higher activity and this higher activity was partly due to spontaneous inputs from VOI_proxy_. There is an apparent discrepancy in our results, showing on the one hand linear summation of spontaneous and stimulus-evoked effects in the BOLD responses, and on the other hand an interaction of these two effects in the behavioural responses to the stimulus. Although the number of participants in our study was modest, we do not believe that this led to the absence of an interaction in the BOLD responses, since we demonstrated very significant main effects of spontaneous and stimulus-evoked inputs. Furthermore, the absence of an interaction in the BOLD response does not allow one to infer that spontaneous fluctuations do not influence stimulus processing at a neuronal level (for example, a significant interaction at the neuronal level may be suppressed by saturating non-linearities in the hemodynamic response function). These nonlinearities may differ between brain areas, and might therefore also explain differences between studies; it is possible that no interaction at the level of hemodynamic responses is observed in primary sensory and motor areas such as M1 (Fox et al., 2006) and V1 (Bianciardi et al., 2009), but that spontaneous and task-related effects interact in a nonlinear way in higher-level areas such as the FFA (Hesselmann et al., 2008a) and V5/MT (Hesselmann et al., 2008b). Furthermore, our measurements were conducted using a low-contrast stimulus; however, stimulus contrast affects certain aspects of stimulus processing such as the relative contribution of feed-forward versus lateral connections (Nauhaus et al., 2009); if such differences in neuronal processing translate into differences in the stimulus-evoked BOLD response, our results might have been different at higher stimulus contrasts.

### Spontaneous fluctuations and response variability

We found that spontaneous fluctuations accounted for a large portion of the variability in the BOLD signal during visual responses, in both striate and extrastriate areas. This result is in agreement with findings in the motor cortex, where spontaneous activity contributes substantially to the variability in fMRI activity during button presses (Fox et al., 2006, 2007). Taken together, these results suggest that spontaneous fluctuations have similar effects throughout the cerebral cortex, and therefore that these findings are of general relevance.

In addition to contributing to BOLD response variability, spontaneous fluctuations also seemed to predict behavioural responses to the stimulus. Activity in V1 reflected our participants' performance; the BOLD response to the stimulus was slightly higher on correct compared to incorrect trials, in agreement with previous findings (Ress et al., 2000; Hesselmann et al., 2010). Conversely, in 4 out of 6 participants, trials with high levels of spontaneous activity were associated with a higher probability of yielding a correct response. This influence of spontaneous fluctuations on behaviour is consistent with their role in previously observed BOLD–behaviour relationships. For example, 74% of the variability in force that is normally observed when participants are required to press a button repeatedly, can be accounted for by spontaneous activity in the motor cortex (Fox et al., 2007). The discrepancy between this large effect of spontaneous activity on variability in motor output, and the modest effect on visual perception that we find here, might be explained by the fact that such small activity differences in visual perception between correct and incorrect trials are usually found after averaging over no fewer than several hundred (Ress et al., 2000), or even thousand (Ress and Heeger, 2003) trials. While our study collected a large amount of data, the necessary spacing between trials in our design limited the number of trials overall. Our small effect sizes could therefore be due to a lack of statistical power.

Our findings are also consistent with the recent proposal that the influence of spontaneous fluctuations on the processing of incoming stimuli can be understood in terms of a predictive coding account of neuronal activity (Hesselmann et al., 2010; Sadaghiani et al., 2010). This theory suggests that brain activity is governed by the need to minimise surprise (Friston, 2010). This can be achieved by continuously comparing incoming sensory input to an internal model that generates top-down predictions of that input. Optimal prediction rests on two distinct processes; first, inferring the content of a percept, and second, inferring the uncertainty or precision of that prediction. Critically, activity in sensory cortex reflects both of these processes. Under this account of stimulus processing, fluctuating levels of spontaneous activity may reflect fluctuations in the precision of the sensory input. High spontaneous activity may confer a greater precision onto (bottom up) sensory inputs, leading to a correct perceptual decision. This is in agreement with our findings (Fig. 5).

### Cognitive processes underlying spontaneous fluctuations

The activity in primary visual cortex recorded by fMRI represents a complex interplay between feed-forward stimulus-induced activity, feedback activity from other visual areas, and endogenous factors such as attention and arousal. In our analysis, we have sought to separate this myriad of influences into stimulus-evoked and spontaneous inputs into our stimulus-sensitive region. An important factor contributing to the spontaneous input into a V1 region is undoubtedly attention. Attention enhances neuronal activity in parts of the visual cortex corresponding to an attended location (Brefczynski and DeYoe, 1999; Kastner et al., 1999), and influences participants' reaction times and accuracy, as is demonstrated in the classic Posner paradigm (Posner, 1980). Moreover, spatial attention causes an additive shift in V1 activity that enhances activity prior to the stimulus as well as the stimulus-evoked response (Sylvester et al., 2009). It is not surprising, therefore, that many BOLD–behaviour relationships have been attributed to fluctuations in attention (Pessoa et al., 2002; Ress et al., 2000; Sapir et al., 2005). Indeed, the encoding of the precision of sensory signals by optimising synaptic gain (Friston, 2010) is entirely consistent with mechanistic notions of attention gain (Reynolds and Heeger, 2009).

**Supplementary Fig. 7:**
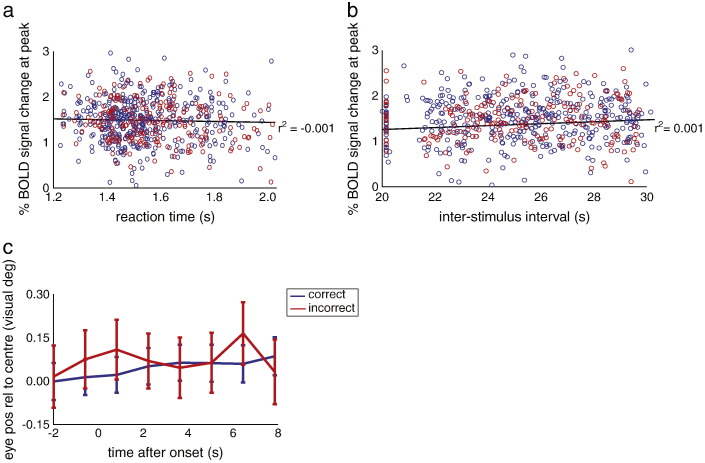
Other factors influencing perception and response variability. (a) Reaction time did not correlate with the peak activity of the evoked BOLD responses, F(5,280) = 0.506, p = 0.477. (b) Inter-stimulus interval length did not correlate with the peak activity of the evoked BOLD responses, nor did it affect the perceptual outcome or reaction time of the subsequent trial (F(5,280) = 0.343, p = 0.558). (c) Eye position did not differ between correct and incorrect trials at any time during presentation of the stimulus (t(383) < 0.23, p > 0.45; eye position across all participants, error bars reflect standard error of the mean).

Our design lacked an explicit manipulation of attention, and therefore we are unable to assess the contribution of attention per se to the observed variability in the BOLD and behavioural responses to the stimulus. However, we argue that attention cannot have been the sole mechanism responsible for the observed response variability. First of all, our proxy for spontaneous inputs was based on distributed voxels not constituting a single anatomical region and is therefore unlikely to reflect purely attentional top-down effects, yet was still able to account for a substantial fraction of the variance in the BOLD signal. Second, reaction times, which can be used as a marker for attention (Posner, 1980), did not correlate with the peak amplitude of the BOLD response (Supplementary Fig. 7a). This implies that any putative lapses in attention during stimulus presentation, owing to its long duration (1 s), did not influence the variability in the subsequent BOLD response. Furthermore, there was no significant relationship between inter-stimulus interval (ISI) length and the peak of the BOLD response (Supplementary Fig. 7b), arguing against increased stimulus expectancy (reflected in a larger BOLD response) for long ISIs. Also, although ISIs were variable, their length did not affect task performance (neither accuracy nor reaction time, see legend Supplementary Fig. 7b). This suggests participants maintained their attention quite steadily, at least during the final part of the ISI (since the ISI was always 20–30 s, participants could have adopted a strategy of only paying attention towards the end of an ISI). Lastly, eye position was not significantly different for correct and incorrect trials during presentation of the stimulus (Supplementary Fig. 7c).

## Conclusion

In conclusion, we show that a local region in V1 was influenced by both stimulus-driven and spontaneous fluctuations in input (with little evidence for an interaction between these components). Crucially, the resulting spontaneous fluctuations were responsible for a large component of both neuronal and behavioural variability in response to stimuli.

The following are the supplementary materials related to this article.

Supplementary Table 1 Location of VOI_proxy_ voxels. MNI coordinates for the three main clusters (all > 10 voxels) of VOI_proxy_ voxels are shown for each participant.

Supplementary Table 2 Individual participants' behaviour. For each participant, the total number of trials, D-prime, and reaction time for correct and incorrect trials are shown.

Supplementary Table 3 Changes in signal power, noise power, and SNR per participant after subtracting the spontaneous activity. Asterisks indicate significance. Note that the change in signal power (for individual participants) represents a single value and can therefore not be statistically assessed.

Supplementary materials Supplementary methods and figures

## Figures and Tables

**Table 1:** Key statistics of the main effects and their interaction. FWE-corrected p-values and associated Z-scores are shown for the main effect of stimulus, spontaneous input, and their interaction, for the cluster within a sphere of 8 mm radius centred on the V1 voxel that showed the highest response to the localiser stimulus, at MNI coordinates [2 –78 − 8]. Cluster size in number of 3x3x3 mm voxels, peak voxel in [x y z] coordinates in MNI space.

Contrast	Z-score	p-value	Cluster size	Peak voxel
Stimulus	3.41	0.024	9	[4 −80 − 6]
Spontaneous	3.30	0.036	4	[0 − 76 − 6]
Interaction	1.23	0.451	0	[2 − 78 − 8]
