# Supplementary material for: The influence of spontaneous activity on stimulus processing in primary visual cortex
Source: Neuroimage. 2012 Feb 1;59(3-2):2700–8. doi: 10.1016/j.neuroimage.2011.10.066 (PMC3382731; doi:10.1016/j.neuroimage.2011.10.066)
Supplement: Supplementary Table 1 — Location of VOIproxy voxels. MNI coordinates for the three main clusters (all > 10 voxels) of VOIproxy voxels are shown for each participant. [file mmc1.doc]

| **participant** | **MNI coordinates of 3 major clusters** | | |
| --- | --- | --- | --- |
| s1 | [ 4 -58 -14] | [-20 -58 -10] | [-46 27 27] |
| s2 | [ -8 -54 -13] | [-16 -46 -18] | [-42 32 -18] |
| s3 | [ 9 -57 -11] | [-16 -52 -15] | [-18 -63 5] |
| s4 | [ 7 -47 8] | [-18 -69 -27] | [-32 -69 -23] |
| s5 | [-7 -77 -20] | [-29 -61 -25] | [-15 -69 -15] |
| s6 | [ 5 -69 -47] | [ -5 -80 -47] | [ 5 -67 -18] |
