# Supplementary material for: The influence of spontaneous activity on stimulus processing in primary visual cortex
Source: Neuroimage. 2012 Feb 1;59(3-2):2700–8. doi: 10.1016/j.neuroimage.2011.10.066 (PMC3382731; doi:10.1016/j.neuroimage.2011.10.066)
Supplement: Supplementary Table 2 — Individual participants' behaviour. For each participant, the total number of trials, D-prime, and reaction time for correct and incorrect trials are shown. [file mmc2.doc]

| **participant** | **nr trials** | **D-prime** | **RT correct** | **RT incorrect** |
| --- | --- | --- | --- | --- |
| s1 | 119 | 1.11 | 1457 ms | 1467 ms |
| s2 | 117 | 0.83 | 1716 ms | 1747 ms |
| s3 | 141 | 1.22 | 1719 ms | 1781 ms |
| s4 | 130 | 1.21 | 1634 ms | 1696 ms |
| s5 | 139 | 1.25 | 1506 ms | 1540 ms |
| s6 | 122 | 0.76 | 1679 ms | 1729 ms |
