# Supplementary material for: The influence of spontaneous activity on stimulus processing in primary visual cortex
Source: Neuroimage. 2012 Feb 1;59(3-2):2700–8. doi: 10.1016/j.neuroimage.2011.10.066 (PMC3382731; doi:10.1016/j.neuroimage.2011.10.066)
Supplement: Supplementary Table 3 — Changes in signal power, noise power, and SNR per participant after subtracting the spontaneous activity. Asterisks indicate significance. Note that the change in signal power (for individual participants) represents a single value and can therefore not be statistically assessed. [file mmc3.doc]

| **participant** | **change in signal (%)** | **change in noise (%)** | **change in SNR (%)** |
| --- | --- | --- | --- |
| s1 | -2.0 | -25.5* | 31.5* |
| s2 | 9.2 | -20.3* | 37.0* |
| s3 | -1.2 | -16.0 | 17.6 |
| s4 | 0.0 | -25.4* | 34.0* |
| s5 | -18.0 | -33.9* | 24.0 |
| s6 | 1.6 | -31.8* | 48.9* |
| **ALL** | **-1.7** | **-25.5*** | **32.2*** |
