# Supplementary material for: The influence of spontaneous activity on stimulus processing in primary visual cortex
Source: Neuroimage. 2012 Feb 1;59(3-2):2700–8. doi: 10.1016/j.neuroimage.2011.10.066 (PMC3382731; doi:10.1016/j.neuroimage.2011.10.066)
Supplement: Supplementary materials — Supplementary methods and figures [file mmc4.doc]

## Supplementary Material

**Supplementary Methods**

### Retinotopic mapping and ROI localiser

Each participant completed two successive 10 minute scanning runs of a conventional retinotopic mapping procedure, viewing a contrast-reversing, black-and-white checkerboard stimulus presented in alternating 15 second blocks as wedges covering either horizontal or vertical meridians for V1, V2, and V3 localisation (Sereno et al., 1995, Teo et al., 1997, Wandell et al., 2000). The retinotopic representation of the stimulus location in the visual field in the early visual areas was determined in two 6-minute long scanning runs by measuring brain activity while participants viewed black-and-white checkerboard stimuli (at the stimulus location and its mirror locations in the other hemisphere and in both lower visual fields) flickering at 10 Hz on a grey background. The checkerboard stimuli were circles of identical size and eccentricity as the stimulus used in the actual experiment. Alternating 15 second periods of the left-upper and right-lower, versus the right-upper and left-lower stimulus, were shown for 6 minutes per run. To attract attention to the stimuli, participants were required to press a button as soon as a small red dot flashed in any stimulus throughout these runs, while they fixated on the central fixation dot.

ROI data were analysed using SPM5 ([www.fil.ion.ucl.ac.uk/spm/software/spm5/](http://www.fil.ion.ucl.ac.uk/spm/software/spm5/)). In addition to nuisance regressors modelling participant movement and physiological parameters (see above), the blocks of checkerboard stimuli in the left-upper and right-lower, and the right-upper and left-lower visual field were modelled as regressors. These regressors were convolved with a synthetic hemodynamic response function and entered into a General Linear Model (GLM), which produced activation maps of the four ROI localiser regions. The current analysis focused on activity in the ROI representing the stimulus location (left upper visual field); the three other ROIs were not used in the present analysis. Retinotopic cortical areas were identified using Freesurfer (http://surfer.nmr.mgh.harvard.edu/). This yielded maps of functionally defined visual areas V1, V2, and V3 for each participant. These maps were combined with the ROI activation images to reveal retinotopic regions in V1, V2, and V3 representing the spatial location of the stimulus in the actual experiment (ROI_stim_).

### Supplementary References

Sereno, M.I., Dale, A.M., Reppas, J.B., Kwong, K.K., Belliveau, J.W., Brady, T.J., Rosen, B.R., Tootell, R.B., 1995. Borders of multiple visual areas in humans revealed by functional magnetic resonance imaging. Science 268, 889-893.

Teo, P.C., Sapiro, G., Wandell, B.A., 1997. Creating connected representations of cortical gray matter for functional MRI visualization. IEEE Trans Med Imaging 16, 852-863.

Wandell, B.A., Chial, S., Backus, B.T., 2000. Visualization and measurement of the cortical surface. J Cogn Neurosci 12, 739-752.

**Supplementary Tables**

**Supplementary Table 1** **Location of VOI_proxy_ voxels.** MNI coordinates for the three main clusters (all >10 voxels) of VOI_proxy_ voxels are shown for each participant.

**Supplementary Table 2** **Individual participants’ behaviour.** For each participant, the total number of trials, D-prime, and reaction time for correct and incorrect trials are shown.

**Supplementary Table 3** **Changes in signal power, noise power, and SNR per participant after subtracting the spontaneous activity.** Asterisks indicate significance. Note that the change in signal power (for individual participants) represents a single value and can therefore not be statistically assessed.

**Supplementary Figures**

**Supplementary Figure 1 Location of VOI_proxy_.** The location of the 100 voxels best correlated to the stimulus region during rest is shown for all participants. VOI_proxy_ voxels are in red and encircled with white broken lines, the location of ROI_stim_ is in blue and encircled with a solid line. Outlines of visual areas V1, V2, and V3 are shown on each hemisphere with light yellow broken lines. Labels belonging to these visual areas are shown on the left hemisphere of one participant showing no VOI_proxy_ voxels; these labels hold for all (left) hemispheres shown and are mirrored in the opposite (right) hemispheres. In some participants, visual areas are split up by the way the cortex is cut and flattened. The one hemisphere showing no VOI_proxy_ voxels also shows several anatomical landmarks. STS, superior temporal sulcus. IPS, inferior parietal sulcus. LH, left hemisphere. RH, right hemisphere.

**Supplementary Figure 2 Stability of coupling between VOI_proxy_ and ROI_stim_. (a)** The time course of ROI_stim_ during the rest run for one participant is shown together with the average time course of VOI_proxy_. The voxels of this VOI_proxy_ and their weights were chosen such as to maximise the correspondence with the time course of ROI_stim_, as explained in the main text. **(b)** The time course of ROI_stim_ during another rest run in the same participant, collected on a different day. The same voxels and weights were used to construct the average time course of VOI_proxy_, and it is evident that there is still a close correspondence between the two time courses, confirming the stability of the coupling between ROI_stim_ and VOI_proxy_ over time. In **(a)**, the activity in VOI_proxy_ explained 93.7% of the variance in ROI_stim_, in **(b)** this was 46.4%.

**Supplementary Figure 3 Interaction effect size.** Posterior probability maps show all voxels whose activation by the interaction of spontaneous and stimulus-induced inputs exceeds, with 95% probability, a threshold of 0 % BOLD signal change **(a)**, 0.2 % BOLD signal change **(b)**, or 0.7 % BOLD signal change **(c)**. As can be readily observed, the interaction effect in the stimulus region in V1 (red circle), if any, is extremely small.

**Supplementary Figure 4 Effect of spontaneous activity on BOLD response variability in V2 and V3. (a,e)** The raw BOLD response is plotted for all trials of the same representative participant as in Fig 3. **(b,f)** The spontaneous input measured in separately computed VOI_proxy_’s for V2v and V3v. **(c,g)** Subtracting the spontaneous input reduced the variability in the evoked BOLD responses. **(d,h)** Signal power (left), noise power (middle), and SNR (right) in V2v and V3v for all participants before (‘raw’; in blue) and after (‘corr’; in red) subtracting the estimated spontaneous activity. The y-axis on the left in both graphs corresponds to the signal and noise power, the y-axis on the right to the SNR. Noise power was significantly reduced in both cases, yet SNR not significantly increased, after spontaneous activity subtraction. For details, see Fig 3.

**Supplementary Figure 5 BOLD responses associated with four response categories.** Dividing the trials into hits, misses, false alarms, and correct rejections did not reveal any differences in BOLD activity, either before **(a)** or after **(b)** subtracting spontaneous activity.

**Supplementary Figure 6 BOLD responses to the stimulus.** BOLD responses were similar for those trials where the grating was present versus absent. Note that the circular patch of random grey noise was present in both cases, giving rise to the robust BOLD response even when the grating was absent.

**Supplementary Figure 7 Other factors influencing perception and response variability. (a)** Reaction time did not correlate with the peak activity of the evoked BOLD responses, F(5,280) = 0.506, p = 0.477. **(b)** Inter-stimulus interval length did not correlate with the peak activity of the evoked BOLD responses, nor did it affect the perceptual outcome or reaction time of the subsequent trial (F(5,280) = 0.343, p = 0.558). **(c)** Eye position did not differ between correct and incorrect trials at any time during presentation of the stimulus (t(383) < 0.23, p > 0.45; eye position across all participants, error bars reflect standard error of the mean).
